# Cerebrospinal fluid dynamics and intracranial pressure elevation in neurological diseases

**DOI:** 10.1186/s12987-019-0129-6

**Published:** 2019-04-10

**Authors:** Steven William Bothwell, Damir Janigro, Adjanie Patabendige

**Affiliations:** 1 Brain Barriers Group, School of Biomedical Sciences and Pharmacy, The University of Newcastle, Medical Sciences Building, University Drive, Callaghan, NSW 2308 Australia; 2 FloTBI Inc., Cleveland, OH USA; 3 Department of Physiology, Case Western Reserve University, Cleveland, OH USA; 4 Hunter Medical Research Institute, Newcastle, NSW Australia; 5 The Institute of Infection and Global Health, University of Liverpool, Liverpool, UK

**Keywords:** Cerebrospinal fluid dynamics, Intracranial pressure elevation, Choroid plexus, Blood–brain barrier, Ischaemic stroke

## Abstract

The fine balance between the secretion, composition, volume and turnover of cerebrospinal fluid (CSF) is strictly regulated. However, during certain neurological diseases, this balance can be disrupted. A significant disruption to the normal CSF circulation can be life threatening, leading to increased intracranial pressure (ICP), and is implicated in hydrocephalus, idiopathic intracranial hypertension, brain trauma, brain tumours and stroke. Yet, the exact cellular, molecular and physiological mechanisms that contribute to altered hydrodynamic pathways in these diseases are poorly defined or hotly debated. The traditional views and concepts of CSF secretion, flow and drainage have been challenged, also due to recent findings suggesting more complex mechanisms of brain fluid dynamics than previously proposed. This review evaluates and summarises current hypotheses of CSF dynamics and presents evidence for the role of impaired CSF dynamics in elevated ICP, alongside discussion of the proteins that are potentially involved in altered CSF physiology during neurological disease. Undoubtedly CSF secretion, absorption and drainage are important aspects of brain fluid homeostasis in maintaining a stable ICP. Traditionally, pharmacological interventions or CSF drainage have been used to reduce ICP elevation due to over production of CSF. However, these drugs are used only as a temporary solution due to their undesirable side effects. Emerging evidence suggests that pharmacological targeting of aquaporins, transient receptor potential vanilloid type 4 (TRPV4), and the Na^+^–K^+^–2Cl^−^ cotransporter (NKCC1) merit further investigation as potential targets in neurological diseases involving impaired brain fluid dynamics and elevated ICP.

## Background

Elevation of intracranial pressure (ICP) after a neurological injury has been reported in numerous conditions including hydrocephalus, idiopathic intracranial hypertension (IIH), oedema, traumatic brain injury (TBI), and stroke [1]. Uncontrolled raised ICP can worsen outcomes, and several manoeuvres have been proposed to mitigate ICP elevations. The deleterious consequences of unchecked ICP highlight the importance of maintaining ICP homeostasis within the central nervous system (CNS). Cerebrospinal fluid (CSF) is an important component of maintaining a stable ICP, and disruptions to secretion or drainage can lead to ICP elevations [2]. However, a cogent review of the involvement of CSF in the elevation of ICP in pathologic conditions of the CNS is currently lacking.

CSF serves as a protective fluid to the brain and spinal cord, cushioning them from mechanical injury, and acts to reduce the brain’s effective weight—its actual mass is ~ 1500 g while the buoyancy provided by CSF reduces its net weight to 25–50 g [3]. It serves as a critical mechanism for transporting nutrients and hormones from one area to another [3–5]. CSF also plays a role in protein clearance within the CNS by mechanisms hotly debated [6–9]. Reduced CSF secretion as a function of aging [1] contributes to increased protein aggregation and has links to increased beta-amyloid deposition in Alzheimer’s disease [10, 11] or phosphorylated tau protein in chronic traumatic encephalopathy [12].

**Fig. 1:**
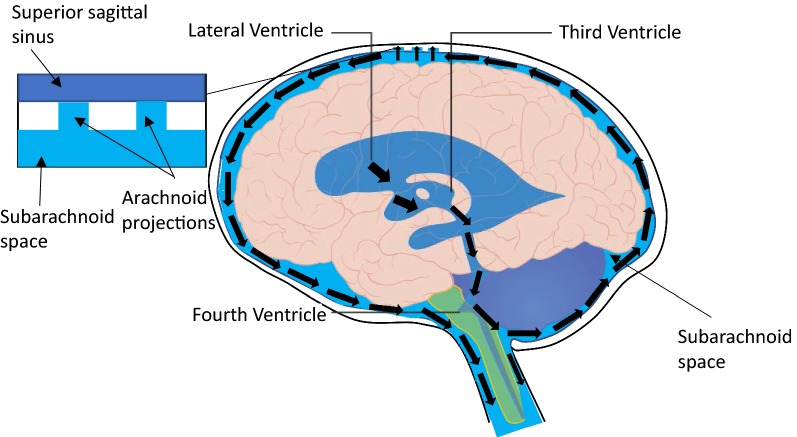
Conventional view of CSF flow. CSF is produced in the ventricles, beginning in the lateral ventricles, and flows toward the subarachnoid space. CSF circulates in the subarachnoid space and drains into the subarachnoid space (via arachnoid projections) and the spinal cord. Arrows depict the flow of CSF from the lateral ventricle origin

The exact mechanisms of CSF secretion, flow and reabsorption/drainage are debated; however, it is understood that alterations to normal physiology can contribute to elevated ICP. The traditional view of CSF drainage is that fluid flows from the subarachnoid space through the arachnoid villi and drains into the blood of the superior sagittal sinus (Fig. 1) [13]. Studies conducted in multiple animal species also point to lymphatic drainage of CSF in which CSF exits the cranium through the cribriform plate and spinal canal to reach the cervical and spinal lymph nodes [14–18]. While there is debate about the dynamics of CSF, studies have attempted to elucidate how our current hypotheses of CSF physiology may be influenced by neurological disorders or injury. Factors influencing secretion, flow and drainage are under investigation and are relevant in conditions of elevated ICP, as CSF contributes to the overall pressure of the CNS. An assessment of how both ICP and CSF physiology are affected by pathology may offer us a potential target for attenuating ICP elevations in several conditions of the CNS.

This review aims to examine existing literature—acknowledging our current understanding of CSF physiology and controversies in the field, while exploring how altered secretion and drainage may link to ICP in pathology. This will allow us to identify gaps in knowledge and to identify new targets for research within the field of CNS injury.

## Review

### CSF secretion

#### The choroid plexus as the primary site of CSF secretion

It is estimated that around 80–90% of CSF is secreted by the choroid plexus, a highly-vascularised structure of epithelial cells located in the ventricles of the brain. The epithelial cells surrounding the capillaries compose a blood-CSF barrier (BCSFB), which selectively controls the movement of solutes and water to regulate the composition of CSF [19]. Under normal physiological conditions, the total volume of human CSF is between 150 and 160 mL [20]. To maintain this volume, CSF secretion and drainage must be equal; imbalances to this equilibrium will produce an increase in the total fluid content of the brain, subsequently causing an elevation in pressure.

Some of the first supporting evidence for the role of the choroid plexus in CSF secretion came from Dandy [21], which involved the removal of choroid plexus tissue from one of the lateral ventricles in a canine. In this study, the ventricle containing the remaining choroid plexus expanded while the other shrank. Further evidence includes experiments by Welch et al. which demonstrated a higher haematocrit in the main choroidal vein when compared to the main choroidal artery [22, 23]. These results implied a movement of fluid and solutes (but not blood-borne cells) across the choroid plexus into the ventricles, which we now accept as the foundations for CSF secretion.

When looking to anatomical clues for the involvement of the choroid plexus in CSF secretion, ultrastructure of rat choroid plexus epithelial cells, as revealed by electron microscopy, highly resembles that of typical secretory cells with the presence of: a brush border at the apical membrane facing the ventricle, infoldings at the basolateral membrane, a high density of mitochondria, and a well-developed endoplasmic reticulum [23]. This evidence would point to a secretory role of the choroid plexus.

#### Extra-choroidal fluid secretion

The blood–brain barrier (BBB), formed by endothelial cells that line cerebral capillaries connected by tight junctions, is the site of regulated exchange of ions, molecules and cells between the blood and the brain [24–26]. Fluid transport across the BBB contributes to the remaining 10–20% of CSF secretion [27–29]. Interstitial fluid (ISF) is produced by fluid secretion across the BBB [28, 30]. ISF fills the narrow extracellular space (ECS) between neurons and glia within the parenchyma, while CSF occupies the ventricles and subarachnoid space [31]. The interaction between ISF and CSF is likely to occur in the perivascular spaces (low resistance pathways) surrounding the vessels supplying the brain parenchyma through bulk flow—which is convective in nature. It was previously demonstrated that tracers of different sizes were cleared from the brain at similar rates despite differing diffusion coefficients. These observations imply convective flow, as they are inconsistent with diffusive flow. It is suggested that these tracers, as well as endogenous proteins in the CSF, are distributed around the brain via a combination of convective flow in the perivascular spaces and diffusion in the tortuous brain ECS [31, 32]. Therefore, the purpose of this interaction/exchange between CSF and ISF is thought to be for the distribution of compounds from one brain region to another, like melatonin [33], or for distribution of compounds from the systemic circulation, like vitamin C and folate entering the CNS via the choroid plexus [31, 34]. CSF can flow into these perivascular spaces from the subarachnoid space via pores, termed stomata, in the penetrating arteries [35]. Here, both fluids interact and may flow back into CSF compartments to be drained via the major CSF drainage pathways.

#### Drivers of CSF secretion

Some argue that the generation of CSF is driven by the hydrostatic pressure gradient between the blood, choroid plexus epithelial cells, and the ventricles—according to Starling’s law of filtration [36]. This means that increased ICP, observed in hydrocephalus, may attenuate CSF secretion and, conversely, decreased pressure will increase CSF secretion. The role of pressure gradients in CSF secretion is unclear. If pressure gradients were the primary driver of CSF secretion then this would suggest that it is a fluid derivative of serum, produced from ultrafiltrate, which is contrasted in various studies of CSF regulation in which its ability to independently regulate its ionic milieu has been demonstrated—the composition of ions in CSF differs significantly from that of plasma (Table 1) [37, 38]. Protein content of CSF (0.03 g/dL) is lower than that of plasma (7 g/dL), with a CSF/plasma ratio of 0.004 [37].

**Table 1:** CSF composition reported for human, rabbit and dog

Solute	Human^a^		Rabbit^b^		Dog^b^	
	Plasma Conc.	CSF Conc.	Plasma Conc.	CSF Conc.	Plasma Conc.	CSF Conc.
Na^+^ (mM/L)	153	147	148	149	155	151
K^+^ (mM/L)	4.7	2.9	4.3	2.9	4.6	3.0
Ca^2+^ (mM/L)	1.3	1.1	5.6	2.5	5.7	2.9
Mg^2+^ (mM/L)	0.6	1.1	2.0	1.7	1.4	2.0
Cl^−^ (mM/L)	110	113	106	130	121	133
HCO_3_^−^ (mM/L)	24	22	25	22	26	26
pH	7.40	7.33	7.46	7.27	7.42	7.42
Osmolarity (mOsm)	290	290	298.5	305.2	299.6	305.2

^a^Human CSF values from Ransom [37]

^b^Rabbit and dog CSF values from Damkier et al. [38]

Additionally, alterations in CSF osmolarity have been shown to influence water flux across the choroid plexus and the BBB [39]. This series of experiments was performed by Klarica et al. with the aim of disproving this traditional view of CSF secretion. However, the influence that altered osmolarity exerts on water flux is not contradictory to the currently accepted hypothesis, and in fact, suggests mechanisms of CSF homeostasis that are dependent on osmolarity.

While the ISF is continuous with the CSF, the composition of the former can dramatically differ in ions where neuronal activity occurs. We will present one of the many mechanisms of focal changes in interstitial ionic concentrations that while pronounced in specific extracellular domains have negligible if any effect on overall CSF composition. Another example relates to glutamate “buffering” at the synapse. Focal glutamate and potassium increases have a profound effect on neuronal firing and, if uncontrolled, may cause neurotoxicity. As in the case of extracellular potassium regulation (see below) the local accumulation of glutamate is not readily measurable in ISF/CSF until exaggerated levels are present. The movement of water across the BBB and within the brain parenchyma follows osmotic gradients. A frequent misperception is that water will enter the brain after blood–brain barrier disruption alone [40]. However, since the osmolarity of brain and serum is roughly identical [41], BBB disruption alone may not be sufficient to cause oedema. After BBB disruption, potassium moves down its concentration gradient from blood into the brain resulting in a K^+^ concentration that is sufficient to depolarize neurons, trigger action potentials and drive repolarization further elevating cerebral K^+^ levels. Thus K^+^ homeostasis overlaps with control of water content in the brain. However, when the BBB is disrupted, the capillary endothelium acts similar to fenestrated capillaries. Therefore, both osmotic and hydrostatic pressure gradients contribute to oedema formation. When this happens, ICP and systemic blood pressure, which are determinants of hydrostatic pressure, assume an important role [42].

The concentration of potassium in the ISF in the mammalian brain increases measurably (3–4 mM) during physiologic stimulation, to a larger extent (up to 12 mM) during seizures or direct synchronous stimulation of afferent pathways, and to exceedingly high values (> 30 mM) during anoxia or spreading depression. In spite of these rapid and large changes in extracellular potassium (K_out_), values return to normal levels in a relatively short time. Neuronal excitability is regulated by a complex interaction of excitatory and inhibitory potentials. In pyramidal neurons, depolarising ion conductances involved in fast action potential generation are regulated primarily by the voltage-dependent activation/inactivation properties of Na^+^ and K^+^ channels; in addition, inward Na^+^ and Ca^2+^ currents underlie the generation of excitatory postsynaptic potentials (EPSPs). Termination of these depolarising potentials occurs by the voltage- and calcium-dependent activation of potassium conductances and by the activation of interneurons that release inhibitory neurotransmitters to produce inhibitory postsynaptic potentials (IPSPs); the latter are mediated by postsynaptic activation of chloride and potassium currents. Although excitatory events are under physiologic conditions, relatively independent of modest changes in the driving force for the permeant ions, both repolarising potassium and K-IPSP conductances are critically affected by even modest changes in cell resting potential (resting membrane potential, RMP) and [K_out_]. Because neuronal RMP depends significantly, but not exclusively on [K_out_], the maintenance of homeostatic control for extracellular potassium plays a crucial role in the regulation of neuronal firing. Several mechanisms can explain the rapid clearance of K^+^ from the ISF: passive diffusion through the ISF, active removal by blood flow, and neuronal reuptake. However, these mechanisms alone are not fast enough to account for the rapid K^+^ removal from the ISF seen under experimental conditions. Several lines of evidence suggest that brain glial cells and more specifically astrocytes, support the homeostatic regulation of the neuronal microenvironment. This phenomenon is referred to as spatial buffering of extracellular potassium [43–45].

Local control of ion (and water) homeostasis also plays a role in determining the extent and velocity of cerebrovascular response to neuronal activation. Unlike most other organs, the supply of blood (and to some extent the venous return) is under indirect neuronal control. Whether blood vessels of small calibre (arterioles) receive direct parenchymal neuronal afferent is still under debate, but it is increasingly understood that neuronal activity controls local cerebral blood (CBF) flow by coupling of neuronal action potentials to vessel diameter by products of neural activity. Several mediators are involved, including potassium ions (increased blood flow at concentrations below around 10 mM; vasoconstriction at higher potassium levels [46], nitric oxide [47], H^+^/CO_2_ (e.g., during hypercapnia) [48], or by metabolic signal (e.g. adenosine or ATP [49, 50]). Dysfunction of any or all of these feed-forward mechanisms of neurovascular coupling are involved in a broad range of neurological diseases [51–53].

In addition to local control of CBF, the brain is characterised by an additional “anomaly” when compared to peripheral organs. This brain-specific vascular feature impacts several of the issues currently debated (see “glymphatics” below and [30, 31, 54–57]). In the peripheral vascular system, the management of fluid movement across the capillary wall is achieved by a synergistic combination of transcellular and paracellular pathways. Endothelial cell membranes outside of the brain (except in the circumventricular organs), in addition to being permeable to water and gases, express aquaporin 1 (AQP1) water channels [58]. AQP1 is the dominantly expressed aquaporin in peripheral endothelial cells. However, apart from the endothelium in the kidney, the physiological importance of the expression of AQP1 and high water permeability remains undetermined [59]. The interendothelial clefts, fenestrae, or gaps may be the anatomical substrate of the paracellular pathway.

#### The role of ion transport in CSF secretion

As alterations to osmolarity alter water flux across the choroid plexus, it is no surprise that the transport of ions across the BCSFB is important in the secretion process. In this process, ions are transported from circulating blood into the CSF via their respective transporters while water is likely transported by a combination of a transcellular process of uphill water transport (against an osmotic gradient) by cotransporters (e.g. glucose transporter 1, GLUT1) as proposed by Zeuthen et al. [60, 61] and via the paracellular pathway through tight junctions [62, 63]. AQP1, though highly expressed in the apical membrane of the choroid plexus, [64] its role in water transport is undetermined due to its low expression in the basolateral membrane [65]. The transport of Cl^−^, Na^+^ and HCO_3_^−^ are important in this process, and pharmacological studies in which transporters of these ions are blocked have provided evidence of their role in the secretion process [36, 38]. Although many ion transporters identified in the choroid plexus are involved in CSF secretion, the movement of Na^+^, HCO_3_^−^ and Cl^−^ are some of the most important activities in this process [66]. The mechanisms driving this transport require consideration of transporters at both the basolateral and the apical membrane.

**Fig. 2:**
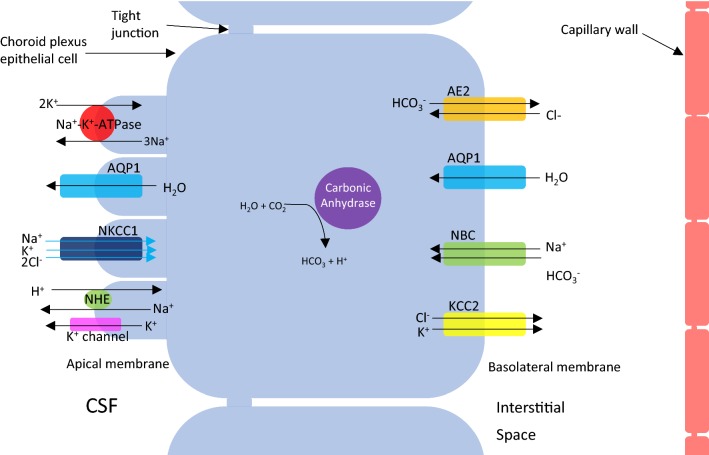
Transepithelial ion transport at the choroid plexus. Movement of solutes from the interstitial space to the intracellular environment via the basolateral membrane are shown—AE2 (epithelial anion exchanger 2), AQP1 (aquaporin 1), NBC (sodium bicarbonate coexchanger) and KCC2 (potassium chloride cotransporter). The movement from the intracellular environment to the CSF in the ventricles via the apical membrane is also shown—Na^+^–K^+^–ATPase, AQP1, NKCC1 (sodium–potassium-chloride cotransporter 1), NHE (sodium–hydrogen exchanger) and K^+^ channel

Ion transport at the choroid plexus epithelial cell is depicted in Fig. 2. Briefly, at the basolateral membrane, the net movement of Na^+^, HCO_3_^−^ and Cl^−^ into the choroid plexus epithelial cell is essential and is driven by the Na^+^ gradient. This gradient is utilised by Na^+^–HCO_3_^−^ cotransporters (NBC) to facilitate the accumulation of HCO_3_^−^ in the epithelial cell. Carbonic anhydrases within the cell also contribute to this accumulation by catalysing the production of HCO_3_^−^ and H^+^ from H_2_O and CO_2_. The resulting HCO_3_^−^ gradient drives Cl^−^ transport into the cell by the epithelial anion exchanger 2, AE2. Simultaneous to the events at the basolateral membrane, transporters at the apical membrane operate to move Na^+^, HCO_3_^−^ and Cl^−^ between the epithelial cell and the CSF. This involves the actions of the Na^+^–K^+^-ATPase pump and inward-rectifying anion currents. The involvement of Na^+^–K^+^–2Cl^−^ cotransporter (NKCC1), expressed in the apical membrane of the choroid plexus is under debate; however, some studies have demonstrated that bumetanide, an NKCC1 inhibitor, reduces CSF secretion at the choroid plexus epithelium [67–70]. Recently, further evidence has emerged to support the involvement of NKCC1 in CSF secretion independently of osmotic driving forces. Steffensen et al. [71] have demonstrated using both ex vivo and in vivo studies on mice that NKCC1 contributes to approximately half of the CSF production by cotransport of water along with its directional translocation of ions independently of an osmotic gradient. In addition, in a rat model of post-haemorrhagic hydrocephalus, NKCC1 was hyper-activated by inflammatory markers in the CSF, and caused bumetanide-sensitive ventriculomegaly [72]. One study proposes that some of the inhibitory actions of bumetanide, a drug thought to act on NKCC1 channels, occur through inhibition of AQP1 at high concentrations. Using *Xenopus laevis* oocytes, they demonstrate that a derivative of the bumetanide compound, AqB013, can inhibit both AQP1 and AQP4 channels with high affinity, confirming the water channel as the site-of-action by targeted mutagenesis [73]. However, a recent study could neither replicate the effect on AQP4-mediated osmotic water permeability by AqB013 and bumepamine nor the bumetanide’s inhibitory action on AQP4 in a rat oocyte assay as reported by Migliati et al. [73]. Yool and colleagues subsequently tested the blocking potential of additional bumetanide derivatives on AQP1 channel conductance and demonstrated attenuation in cancer cell migration upon administration of AqB007 and AqB011 [74]; however, the blocking actions of these derivatives on AQP1 have yet to be demonstrated by other groups, and therefore, their potential actions on CSF secretion remain unknown.

#### Control of CSF secretion

The involvement of carbonic anhydrase in the CSF secretion process has been targeted therapeutically in the treatment of hydrocephalus and IIH with the use of acetazolamide [75]. It acts as an inhibitor of the sulphonamide-sensitive carbonic anhydrases and reduces CSF secretion by ~ 50% [76]. This highlights the importance of HCO_3_^−^ within the choroid plexus epithelium and provides further evidence of the tissue’s role as an important site of CSF secretion. In addition, recent reviews of acetazolamide as a pre-operative strategy for reducing CSF rhinorrhoea and associated elevated ICP are promising, further supporting carbonic anhydrase as a target for attenuating ICP [77].

Investigations of new therapies to reduce ICP in neurological conditions are ongoing. Recently, exendin-4 was studied as a potential modulator of CSF secretion and ICP elevation [78]. Exendin-4 is an agonist of the Glucagon-like peptide-1 receptor (GLP-1R), a class B G-protein coupled receptor, which, upon activation, stimulates adenylate cyclase to convert ATP to cAMP. This increase in cAMP production increases the activation of protein kinase A, which inhibits Na^+^-K^+^-ATPase—an important component of CSF secretion at the choroid plexus. The authors of this study hypothesised that since GLP-1R in kidney cells is already utilised as a target to prevent Na^+^ transport, it may be targeted in a similar way within the choroid plexus to attenuate CSF secretion. Using tissue slices, cell culture and animal models of hydrocephalus, they showed that acute treatment with exendin-4 reduced Na^+^–K^+^-ATPase activity and resulted in a reduction of ICP in female hydrocephalus rats. The authors propose that as GLP-1R agonists are already used in the treatment of diabetes, these drugs could be repurposed for targeting conditions of raised ICP.

#### Aquaporin involvement in CSF secretion

The involvement of aquaporins (AQPs) in fluid production within the CNS has long been established [60, 79]. They are capable of transporting water, ions and solutes across a cell membrane, and therefore, it is likely that they are involved in the process of CSF secretion [80]. AQP1, 4 and 9 are expressed in the brain, with AQP1 and 4 being the main contributors to brain fluid homeostasis. AQP4 is primarily located in the astrocyte processes and endfeet that are located at key interfaces between the brain and major water-containing compartments, such as the BBB, and the BCSFB (at the pial and ependymal surfaces, and possibly fourth and lateral ventricle choroid plexuses, [65]), signifying the importance of AQP4 in regulating brain fluid homeostasis [81, 82]. AQP1 is mainly expressed in the apical membrane of choroid plexus epithelial cells, which suggests a role in CSF secretion. AQPs have the ability to transport water bi-directionally across cell membranes in response to changes in passive osmotic pressure gradients as they have a high capacity and greater selectivity for the water molecules. For example, the selectivity of these channels allows for water to pass through but not acid. AQPs have a narrow pathway that is a very tight fit for water, and the mechanism in the pore allows water molecules to pass through in a single file with no resistance [80, 83]. This mechanism allows AQPs to rapidly transport water across cell membranes. However, several proteins (e.g. NKCC1 and GLUT1) expressed at the endothelium co-transport water independently of osmotic gradients along with their substrates (reviewed in [60]). Therefore, although there is evidence to support a role for AQPs in modulating ISF and CSF circulation, the exact mechanisms and the extent of their contribution remain to be investigated.

A role for AQP1 in CSF secretion seems logical when considering its high expression in the apical membrane of the choroid plexus [64]. Oshio et al. [84] compared choroid plexus water permeability, CSF secretion, and ICP in AQP1-null mouse models with wild-type counterparts to elucidate the contribution of AQP1 to CSF secretion and ICP maintenance. AQP1-null mice showed ~ 20% reduction in the rate of CSF secretion when compared to wild-type mice, accompanied by ~ 50% decrease in ICP. Protein Kinase C activation in both models augmented the difference in secretion rates to ~ 25%. The majority of the decreased ICP in the AQP1-null mice is explained by a drastic reduction in central venous pressure between the two groups; however, when this is accounted for, the authors ascribe ~ 25% of the ICP drop to reduced CSF secretion. These experiments suggest that AQP1 is involved in CSF secretion. However, further studies in multiple animal species are required before a definitive role is assigned to AQP1 in this process.

AQP1 localisation within choroid plexus epithelial cells was shown to be altered in a kaolin-induced hydrocephalus model of mice, in which there was an increase in intracellular vesicle expression of AQP1 coinciding with a decrease in apical membrane expression [85]. These results indicate a compensatory mechanism employed by the epithelium to reduce water flux into the ventricles when there is excessive CSF secretion. Additionally, Wang et al. [85] found a decrease in ventricular size in AQP1-deficient mice, suggesting a reduction in CSF secretion. Overall, these findings implicate AQP1 at the apical membrane in the CSF secretion process, at least in part, and offer a potential target for controlling this process experimentally and therapeutically to target conditions of excess CSF secretion or elevated ICP. In fact, some antagonists of both AQP1 and AQP4 are already under investigation for their translational value in conditions in which these channels are implicated—like hydrocephalus [86].

### CSF flow

From the primary site of secretion in the choroid plexus, CSF flows throughout the ventricular system of the brain. CSF flows from the lateral ventricles to the third ventricle via the foramen of Monro. From here, it flows across the cerebral aqueduct of Sylvius to the fourth ventricle and onto the subarachnoid space through the apertures of Magendie and Luschka [3]. It was previously assumed that CSF flow was driven by pulsations of the choroid plexus and, now, recent research correlates CSF flow through the system with both respiration rate and, to a lesser degree, heart rate [87]. This correlation would observe the bidirectional flow of CSF i.e. rostral movement during deep inhalation and caudal movement during deep exhalation. However, this correlation is challenged by multiple other hypotheses such as CSF flow via the ‘glymphatic’ system (described below) in which CSF moves by convection, and some experiments have demonstrated CSF movement by diffusion [88].

The next step of CSF flow from the subarachnoid space to systemic circulation is under investigation. Many hypothesise transport of CSF into the blood through the superior sagittal sinus, while others argue that CSF drains into external lymphatics. The following section will discuss the available evidence of CSF drainage presented for both direct drainage into the blood and the role of extracranial lymphatics.

### CSF drainage

#### Arachnoid villi

The classical perception of CSF drainage dates back to the eighteenth century and is based on anatomical observations of structures composed of arachnoid cells, appropriately named arachnoid granulations (macroscopic) and arachnoid villi (microscopic). These structures project from the subarachnoid space to the venous sinus within the dura and rely on hydrostatic pressure as the driving force for CSF movement. From here, it is unclear how CSF is transported into the blood; however, some researchers propose that this occurs through gaps in endothelial cells and/or pressure-dependent pinocytosis [89].

The majority of evidence supporting a role for arachnoid villi is anatomical; physiological evidence of their role in CSF drainage is limited. Previous work has demonstrated the movement of CSF through the arachnoid granulations in an in vitro model; however, the exact pressure changes and dynamics occurring within the cranial compartment are difficult to replicate [90].

The appearance of tracer in peripherally circulating plasma following injection into the CSF space suggested that the arachnoid granulations were involved in the transport of CSF across the arachnoid villi [91]. Further, when aiming to investigate the relative contribution of extracranial lymphatic drainage, another hypothesis of CSF drainage, Boulton et al. monitored tracer content in plasma following cervical lymphatic ligation. Their observations led them to the conclusion that the arachnoid villi and cervical lymphatics contributed equally to the clearance of tracer. Additional studies have also implicated the spinal canal in CSF clearance [17]. These observations show that the contribution of the peripheral lymphatic system in tracer transport into plasma must be considered when using tracers to investigate the role of arachnoid villi in CSF clearance.

The appearance of the arachnoid villi occurs after birth. Developmental studies on pre-natal infants have observed a lack of arachnoid villi at this stage [92]. This suggests that alternative mechanisms of CSF drainage are occurring during the pre-natal stage, and it may be that these alternative mechanisms persist in function after birth. Overall, the role of arachnoid granulations in CSF drainage is disputed and it is likely that additional mechanisms also heavily contribute.

The role of arachnoid granulations in CSF drainage may be a secondary mechanism. Injection of ^131^I-human serum albumin into the CSF space of sheep produced an increase in tracer concentration in the intracranial venous sinuses (IVS), which gradually decreased as the concentration of tracer in peripheral venous blood increased (PVB). Further, in animals with a sealed cribriform plate, an elevation in ICP increased the ratio of tracer in the IVS compared to the PVB demonstrating increased uptake of tracer into the intracranial venous sinuses, presumably by the arachnoid granulations. From these observations, the authors proposed a combined model of CSF drainage in which lymphatic exit is the primary site of drainage with the recruitment of arachnoid projections under excessive pressure gradients [18].

#### Lymphatic drainage

Some of the most compelling evidence of CSF drainage mechanisms implicates a role for extracranial lymphatics in CSF clearance. Transport of CSF to the cervical and spinal lymphatics are conserved across several species. CSF flows along the space between the outer dura and the nervous tissue of olfactory and spinal nerves [14, 17, 93, 94]. These observations have given new focus to investigations of CSF drainage, one which has encouraged some researchers to integrate the arachnoid villi hypothesis and others to move away from it completely.

The cribriform plate has been highlighted as an important route of CSF drainage in multiple animal models [14, 16]. Experiments carried out by Johnston’s lab explored the influence of blocking the cribriform plate drainage path on ICP in sheep. By scraping away the olfactory nerves and sealing the cribriform plate with glue, they observed an elevation in ICP that was around double that of animals subjected to sham surgery [14]. Additional studies from the same group used Evans Blue dye and observed accumulation in the lymphatic vessels underlying the cribriform plate following infusion into the CSF space via the cisterna magna [93]. These observations indicate that transport through the cribriform plate via the olfactory nerves is an important mechanism of CSF drainage.

CSF drainage via both the spinal canal and the cribriform plate was also observed in spontaneously hypertensive rats. Using a novel method for contrast-enhanced computed tomography (CT), contrast infused into the lateral ventricles was observed in the spinal canal within 9.1 min and the cribriform plate within 22.2 min [17]. When comparing young and aged rats, it is interesting to note that although ICP was higher in aged animals when compared to young, CSF flow did not differ significantly between the groups. This is surprising since one study previously demonstrated that raised ICP increased CSF transport into the extracranial lymphatics [95]. However, ICP was elevated artificially in the latter study, while the former study looked at natural ICP elevation as a consequence of age. This suggests a homeostatic mechanism for maintaining CSF drainage in aged rats or perhaps the similar drainage rate in aged rats and young rats despite, differences in ICP, is a consequence of reduced CSF secretion in aged rats [96].

#### Continuous fluid exchange

One of the most modern and controversial approaches to CSF dynamics abandons the concept of CSF secretion, flow and drainage at specific loci within the cranial compartment. Instead, it proposes a consistent exchange of fluid between the CSF, ISF, blood and the parenchyma throughout the full system, driven by osmotic and hydrostatic forces [97]. This hypothesis contradicts the large amount of evidence available in support of the ‘classical’ and lymphatics CSF hypotheses, which demonstrate CSF secretion at the choroid plexus and CSF drainage via the arachnoid villi or extracranial lymphatics [89]. One of the main issues of this hypothesis is that water exchange between CSF, ISF and parenchyma is not necessarily indicative of overall CSF movement, as water can move independently of the ions composing CSF. Therefore, water exchange and CSF exchange should be investigated separately. Some have aimed to combine this modern model with the currently accepted model of CSF drainage [98]; however, this viewpoint is far from being accepted by the majority of researchers and further evidence is required for integration of this concept into our current understanding. Another study observed reduced transport of radiolabelled water (H_2_^17^O) following intravenous administration occuring in AQP4 knockout mice but not in the AQP1 knockouts [99]. Overall, the authors of this study used this evidence to argue that CSF secretion is primarily mediated by AQP4 rather than AQP1, therefore, suggesting that the BBB-astrocyte complex plays a more important role in CSF secretion when compared to the choroid plexus.

#### A role of ‘glymphatics’ in CSF drainage

Unlike peripheral organs, the brain does not contain a lymphatics system. Instead, CSF acts as a clearance mechanism for extracellular solutes in the CNS; the exact mechanisms underlying this clearance were previously unclear. Recently, CSF transport along perivascular tunnels, surrounded by astroglial cells, has been described and proposed as a mechanism for protein and solute clearance [7]. This system would support CSF and ISF interaction, as CSF moves from the subarachnoid space into the brain parenchyma along penetrating arteries where it can mix with ISF and drains back into the CSF system along a paravenous pathway. This system appears to be somewhat AQP4-dependent, as deletion of this gene in mice drastically reduces interstitial solute clearance and the clearance of soluble amyloid-β from the CNS. The movement of CSF into the perivascular space of the penetrating arteries is supported by structural investigations with electron microscopy that revealed the presence of specialised pores on the adventitial lining of leptomeningeal vessels within the subarachnoid space—offering a site of entry into the perivascular space [35].

In the context of neurological injury, ‘glymphatic’ function appears to decline. This has been reported in TBI [100], subarachnoid haemorrhage [101, 102] and ischaemic stroke [102]. This impaired function can be detrimental to patients following neurological injury, particularly as the clearance of extracellular proteins becomes impaired. Iliff et al. reported reduced clearance of amyloid-β when ‘glymphatic’ function is impaired in AQP4-absent mice. In 2014, the same group reported impaired tau clearance when ‘glymphatic’ function was reduced following TBI [7, 100]. This attenuation of ‘glymphatic’ function and protein clearance was associated with AQP4 distribution, which was altered post-TBI. Reduction in protein clearance can have significant implications, such as development or early onset of dementia or Alzheimer’s disease in which aggregation of these proteins is reported [103].

It is worth noting that the ‘glymphatic’ hypothesis of protein and metabolite clearance is highly debated. The ‘glymphatic’ hypothesis of CSF movement within the brain involves convective movement of CSF to clear solutes, proteins and metabolites in a process dependent on AQP4 [7]. However, Smith et al. [88] demonstrated movement of fluorescent tracers, which was dependent on size—indicating transport by diffusion. Further, Smith et al. also found that AQP4 knockout mice and rats did not experience any deficiency in protein transport from the subarachnoid space to the brain parenchyma. This hypothesis has also been challenged in other reviews in which the likelihood of convective transport through the brain ECS, in which there is high hydraulic resistance, has been questioned [31]. The perivascular, fluid filled canals surrounding the perforating arteries and veins in the brain parenchyma known as the Virchow–Robin spaces (VRS) allows bidirectional exchange of fluids between the brain ECS and the subarachnoid space. Therefore, the current evidence supports a perivascular fluid system, where fluid flows via convection or dispersion along the perivascular spaces of arteries and diffuses through the neurovascular unit at the capillary level where CSF/ISF exchange is regulated. It then drains out of the parenchyma via perivascular spaces of veins back to the CSF in the subarachnoid space and other sites, including some seepage across the ependymal into the ventricles. This allows for efficient communication between CSF produced by the choroid plexuses and the brain parenchyma.

#### Limitations of current studies

Experiments using protein tracers have been utilised to elucidate the pathways of CSF lymphatic drainage from the cranium; however, attention must be given to the tracers used to ensure that they follow a similar path to that of CSF. Some tracers may be recirculated from plasma to re-enter the lymphatics system, leading to false estimates regarding the extent to which the lymphatic system contributes to CSF drainage [89]. Additionally, the CSF has both an immunological role and an osmolarity regulatory role within the cranial compartment; therefore, separate transport pathways are necessary to control the movement of proteins and metabolites as well as water [3]. This is important to consider in tracer experiments, as the movement of water within the compartment is just as important as protein clearance—particularly when considering the contribution of CSF to elevations in ICP. Additionally, any alterations to CSF osmolarity throughout these experiments could increase water transport into the CSF space and dilute tracer concentrations to give a false indication of tracer transport.

### CSF dynamics and ICP elevation

**Fig. 3:**
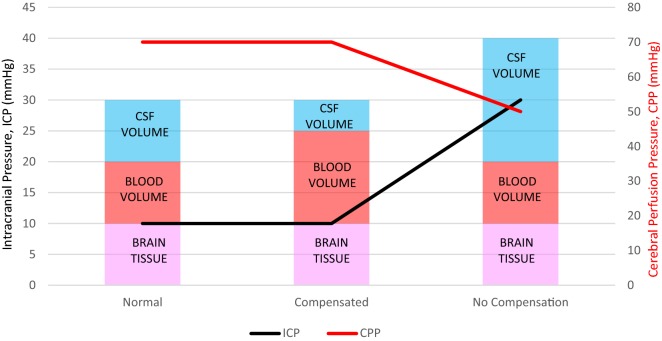
Representation of the relationship between the Monro–Kellie hypothesis, intracranial pressure (ICP) and cerebral perfusion pressure (CPP). Values are based on a baseline mean arterial pressure (MAP) of 80 mmHg. In the compensated model, an increase in cerebral blood volume produces a decrease in cerebrospinal fluid (CSF) volume; this allows ICP and CPP to remain at baseline level. In the model of no compensation, there is no decrease in either cerebral blood volume or brain tissue following an increase in CSF volume; this causes ICP to rise to pathological levels and CPP to decrease in line with the relationship between CPP and ICP (CPP = MAP − ICP)

Changes in CSF can influence ICP. The intracranial compartment within the rigid, non-expandable skull is a closed system consisting of brain, blood and CSF of which the balance between cerebral blood inflow and outflow is essential for maintaining normal ICP. The Monro–Kellie hypothesis dictates that a change in one of the brain, blood or CSF volume will result in the reciprocal change in one or both of the other two. When this is not possible, a further increase in volume will lead to an increase in ICP (Fig. 3) [104]. Acute ICP elevation can reduce cerebral perfusion pressure (CPP), which is determined by subtracting ICP from the mean arterial pressure (MAP). A significant change in ICP can lead to changes in brain perfusion which can alter CPP when autoregulation of cerebral blood vessels is impaired (e.g. during stroke), and chronic conditions of elevated ICP can produce papillary oedema, loss of vision and death [2, 105]. The normal range of ICP in healthy adults is around 5–15 mmHg with increases to 30 mmHg considered pathological, and 40 mmHg life threatening [106]. Current mechanisms of mitigating elevations in ICP often involve invasive surgical intervention. Therefore, investigations of approaches to pharmacologically attenuate these elevations are in high demand [2].

Increased intracranial pressure is reported as a symptom or complication in several CNS pathologies like hydrocephalus [2, 107], IIH [108–110], TBI [111], intracerebral haemorrhage (ICH) [112], subarachnoid haemorrhage (SAH) [26, 113, 114], and ischaemic stroke [115, 116]. A comprehensive review of altered CSF dynamics in each of these CNS disorders would be beneficial; however, the depth required is outside the scope of this review. Instead, this section will briefly outline CSF dynamics in CNS disorders with an extended focus on literature regarding altered CSF dynamics in the context of ischaemic stroke and SAH to reflect the research interests of the authors.

#### Hydrocephalus

Hydrocephalus occurs as a result of several congenital and idiopathic conditions characterised by increased fluid accumulation in the brain and swollen ventricles. Hydrocephalus is caused by disruptions to CSF secretion, flow, or drainage, which lead to increased ICP [2]. Around 95% of hydrocephalus is thought to be caused by disruptions to CSF flow, commonly caused by tumours throughout the ventricular system, and there is some evidence of transient obstructive hydrocephalus as a complication of intraventricular haemorrhage [107]. Hydrocephalus may also be non-obstructive, in which CSF flow within the ventricular system is not impaired but there is decreased absorption. Additionally, tumours of the choroid plexus may also produce increased CSF secretion in rare cases and if this increased secretion is not compensated for by increased outflow, then hydrocephalus can occur [2].

#### Idiopathic intracranial hypertension

A similar condition to hydrocephalus, IIH, describes elevated ICP but without concurrent lesions or CSF disruptions. The incidence of IIH varies worldwide with an estimated incidence of 0.03–2.2 per 100,000, and recent evidence has identified obesity as a major risk factor [108]. The primary treatment of IIH is acetazolamide, which, as discussed above, is a carbonic anhydrase inhibitor capable of reducing CSF secretion. This clinical use of the acetazolamide cleverly targets CSF secretion to decrease the pathological elevation in ICP. However, this seems to be a case of targeting the symptom rather than the cause, and often patients are referred for a ventricular or lumbar CSF shunt [109]. Further, the use of acetazolamide to mitigate the symptoms of IIH may be detrimental in other aspects of CNS health, since CSF has roles in protein and metabolite clearance [110].

#### Traumatic brain injury

Patients suffering TBI often develop oedema, which increases ICP and impairs tissue perfusion. Studies have implicated the role of aquaporins in the oedema development in cases of TBI, and upregulation of AQP4 and AQP9 is observed across the whole brain in experimental animal models [111]. Interestingly, the upregulation of AQP4 and AQP9 correlates with levels of hypoxia inducible factor 1α (HIF-1α), and targeted post-translational down-regulation of HIF-1α by 2-methoxyestradiol reverses the aquaporin upregulation. This demonstrates that in TBI, aquaporin upregulation can be attributed to hypoxia, and is relevant in other conditions of CNS injury, like ischemic stroke, in which hypoxia and oedema also occur.

The result of increased vascular permeability associated with BBB disruption, (or vasogenic oedema) is the paracellular leakage of protein and ion rich fluid into the brain. This can lead to a number of complications after TBI. (1) The increase in ICP from fluid accumulation will cause the intracranial/oncotic pressure to overcome vascular pressure causing blood vessels to collapse and nutrient flow to stop [117]. The first step of this pathological sequela only reduces venous flow, but under condition where ICP ≥ arterial blood pressure, ischemic events occur. (2) Excess extracellular ions and neurotransmitters from the leaky vessels will disrupt the delicate neuronal and glial homeostatic mechanisms which may result in acute post-traumatic seizures. (3) Immunoglobulins, immune cells and inflammatory mediators normally kept out of the immunologically privileged brain now have access to nervous tissue [118]. Conversely, proteins normally sequestered in the brain will then have access to peripheral circulation and tissues [119, 120]. (4) BBB disruption following TBI may prohibit adequate treatment of elevated intracranial pressures with osmotic agents (e.g. mannitol or hypertonic saline) as the gradient which would normally drive water out of the brain might be impaired. There are some recent preclinical studies indicating that modulation of the BBB using small inhibitory RNA directed against claudin-5 may markedly improve the outcome of patients with cerebral oedema [121]. Signs of cytotoxic oedema, like cellular swelling, can be induced in as soon as 30 min following ischaemia, hypoxia and structural injuries. Changes in osmotic balance between the intracellular compartment and the ECS occur as a result of this cytotoxic oedema. This imbalance leads to increased cell volume and a 16% decrease [122] in the volume of ECS. This process leads to a net movement of water from the ECS to the intracellular compartment, and swelling of the brain may occur as a consequence of the ion gradient setup between the ECS and cerebral microvasculature. The gradient induced by depleted Na^+^, water and Cl^−^ promotes ion and water movement across the BBB into the ECS causes an elevation in ICP [42]. The term “ionic oedema” has been assigned to this secondary movement of ions.

#### Intracerebral haemorrhage

In the case of ICH, elevations in ICP are caused by an increased volume entering the intracranial compartment via lesion, independent of CSF. Williamson et al. [112] investigated whether this elevation could be countered by targeting CSF secretion with acetazolamide in a collagenase-induced rat model of ICH. They found that ICH increased ICP in comparison to sham-control rats. Acetazolamide did not attenuate the average ICP in the ICH rats; however, it did reduce the number of transient ICP spikes—characterised by a 1-min peak with an increase of > 20 mmHg. Additionally, ICP was more stable in drug-treated rats—a marker of improved intracranial compliance. In this example, although CSF was not the primary cause of the elevated pressure, it was still beneficial as a target in attenuating the effects of ICH [111]. If CSF can be targeted in this way for ICP disorders that are not a direct result of CSF abnormalities, then it can certainly be used for those in which CSF disequilibrium is the primary cause.

#### Subarachnoid haemorrhage

SAH describes a subset of stroke in which cerebral vessels haemorrhage into the subarachnoid space, accounting for around 5% of strokes [123]. Neurological injury following SAH can be described as biphasic. Global ischaemia and toxicity of subarachnoid blood cause initial brain injury on the incidence of vessel rupture [124, 125]. Delayed brain injury occurs in around 30% of patients and describes delayed neurological deterioration caused by delayed cerebral ischaemia, often presenting up to 2 weeks post-stroke [126]. The predicted long-term outcomes of SAH are worse than that of ischaemic stroke, and many survivors continue to experience cognitive deficits, decreased quality of life, altered mood and fatigue even years after the incident [127]. Secondary physiological responses to SAH can have drastic consequences on the survival of tissue within the brain parenchyma. There is significant evidence of hydrocephalus, vasospasm and increased ICP in response to vessel rupture [112, 113].

A recent study investigated the association between ICP after SAH and clinical outcomes in patients. This was a retrospective study of 116 patients suffering severe SAH, comatose as a consequence of SAH and/or presenting with hydrocephalus post-insult. The authors found that 81% of patients experience at least one episode of elevated ICP (defined as ICP > 20 mmHg for 5 min), and 36% had a ‘highest mean ICP’ of > 20 mmHg (highest mean ICP over a 12-h recording period). When examining the clinical implications of this high ICP, they found an association between high mean ICP (> 20 mmHg) and neurological status, rebleeding, and early lesions detected by CT scan [128]. This would suggest that elevated ICP following SAH leads to secondary degeneration and increased risk. However, as the study was conducted in patients, a causative relationship has not been tested.

Others have also reported increases in ICP following vessel rupture, along with hydrocephalus and vasospasm [112, 113, 129, 130]. A clinical study of 27 SAH patients found that all patients within their cohort experienced some degree of ICP elevation following vessel rupture [26]. Further, this rise in ICP had a negative influence on the CBF. The mechanisms driving this ICP could be attributed to blood entry from the ruptured vessel or oedema development in response to injury. Overall, the rise in ICP observed in patients was associated with poor clinical outcome; therefore, investigations of therapies to target this elevation in ICP are required.

The involvement of CSF in elevated ICP following SAH is yet to be fully defined. However, some clinical observations have reported disruptions to CSF flow associated with SAH [131–134], and studies of animal SAH models support these observations [101, 135]. Disruption of CSF flow along the ‘glymphatic’ pathway was shown following SAH by comparing the distribution of tracers injected into the cisterna magna; this disruption was sustained for at least 4 days with improved distribution at 30 days [101]. The authors proposed that this attenuation in CSF flow was tissue-factor (TF) dependent; however, a causative relationship could not be determined as increased haemorrhaging in the presence of TF antibodies dampened the fluorescent signal of the tracers. Additionally, ICP was increased within 1-min of SAH, reaching a maximum of 290% of the baseline. The elevation was reduced but sustained at 1-h post-stroke, at 151% of the baseline.

Increased ICP following SAH presents patients with additional risks and may contribute to delayed brain injury. Reports of impaired CSF flow following SAH provide insight into the mechanisms underlying this ICP rise and create new pathways for exploring therapeutic tactics to reduce this ICP in the hope of preventing secondary degeneration. One study has already examined the use of tissue plasminogen activator (tPA) in preserving CSF flow post-SAH and found that intracisternal injection of recombinant tPA was able to lower ICP, increase cortical blood volume and partially restore CSF flow 24 h after SAH [135]. A better understanding of CSF circulation and outflow pathways would further advance efforts to minimise deterioration after SAH.

#### Ischaemic stroke

ICP elevation has been demonstrated following ischaemic stroke in both animals and humans [114, 115, 136]. This elevation can have severe consequences such as reduced CBF, BBB disruption and altered fluid movement [38]. These observations have long been associated with oedema development; however, oedema may not be the sole determinant of increased ICP.

Oedema is a known complication of ischaemic stroke, particularly in cases of large cerebral infarction. A higher degree of neurological deficit is reported in stroke patients presenting with oedema when compared to those without [137]. In some cases, this oedema requires medical intervention (mannitol, diuretics, corticosteroids, barbiturates and surgical decompression) to alleviate the consequently high ICP [138]. The attenuation of oedema volume in a rodent model of ischaemic stroke improves functional outcome, which offers a potential target for improving symptoms in humans [139]. Human studies of ICP elevations following ischaemic stroke primarily investigate patients with large oedema, and because of the invasive procedures involved, the studies do not include patients suffering smaller strokes [140]. This leaves us with questions surrounding elevations in ICP in patients suffering smaller strokes: is oedema, in fact, the underlying cause; and if not, what is?

A recent study using a preclinical rodent model of ischaemic stroke demonstrated a transient ICP elevation approximately 24 h after ischaemic stroke [17]. This investigation provides evidence of ICP elevation after relatively minor stroke. Oedema was observed in the ipsilateral hemisphere of the experimental group, which could be prevented by therapeutic short-term moderate hypothermia (32.5 °C). However, volume of oedema could not be correlated with elevations in ICP, suggesting that oedema was not the only cause of ICP elevation in these animals [115]. This study casts doubt on the causative relationship of oedema and ICP that was previously assumed. It is highly likely that a change in CSF volume could be a contributing factor to these observations when there is no change in cerebral blood volume. So how might CSF secretion be altered following ischaemic stroke? Ischaemic stroke is a major stressor in the CNS and can drastically alter the physiology of individual cells, tissue and fluid transport. As discussed above, the choroid plexus is regarded as a major site of CSF secretion; therefore, alterations to its function as an outcome of ischaemic stroke may have an impact on CSF secretion and consequently, ICP.

Reduced blood supply to the lateral ventricle choroid plexus (LVCP) was observed in two rat models of cortical ischaemic stroke: two-vessel occlusion + hypotension model; and, the commonly used, middle cerebral artery occlusion (MCAo) model. Ennis and Keep (2006) found a reduction in LVCP blood flow to 13% of control blood flow with the former model and 62% with the latter. They also investigated the influence of two-vessel occlusion + hypotension on BCSFB integrity by observing the permeability of the barrier with [^3^H]-inulin entry into the CSF. At 6-h of reperfusion, they found that 10 min of occlusion doubled the BCSFB permeability, while 30 min of occlusion trebled permeability [141]. The influence of ischaemia on BCSFB integrity may be significant in understanding how fluid exchange at the choroid plexus alters following ischaemic stroke. However, permeability of the BCSFB was not investigated following MCAo, therefore, it is unclear if this model produces a significant loss of barrier integrity or whether drastic major occlusion is necessary for a noticeable difference. We do know that MCAo causes loss of integrity of the BBB, which can increase fluid, ion and lymphocyte entry into the brain [142].

Ennis and Keep [141] also noted oedema of the choroid plexus after 24 h of permanent occlusion. Additional morphological studies have identified swelling of the choroid plexus epithelium and markers of proliferation through bromodeoxyuridine staining post-stroke [143]. Before that study, no morphological changes were identified by Nagahiro et al. (1994) at 6-h post-reperfusion; however, the difference in observations may result from the different time points observed [144]. Further study of morphological changes to the choroid plexus during and after ischaemic stroke are warranted and may give us more of an understanding of how CSF secretion is affected by ischaemia and an indication of how long disruptions to CSF physiology persist.

Recently, a nonselective cation channel has piqued interest within research of the choroid plexus. Preston et al. [145] have identified that activation of the transient receptor potential vanilloid 4 (TRPV4), a mechano- and osmotic-sensitive channel, can increase ion flux and conductance—a marker of permeability—in a porcine choroid plexus cell line. Activation of the channel allows Ca^2+^ entry into the choroid plexus epithelial cells and results in activation of Ca^2+^ dependent ion channels, specifically the Ca^+^-activated intermediate conductance K^+^ channel, KCNN4c. The authors of this study postulate that the channel plays a role in the regulation of CSF secretion.

This suggestion that TRPV4 is involved in the regulation of CSF is interesting considering other groups have detected an upregulation of TRPV4 in the ipsilateral hemisphere following ischaemia which contributes to greater neuronal injury [146]. Some investigators have even targeted the TRPV4 channels in mice to successfully alleviate cardiac ischaemia/reperfusion injury [147]. Although highly expressed in choroid plexus epithelial cells, as far as we are aware, the expression and activity of TRPV4 in the choroid plexus has not been investigated following ischaemic stroke. Given the results presented by other groups, it would be an interesting path of exploration—particularly considering its potential role in CSF secretion regulation.

The role of aquaporins in CSF secretion was described above, and AQP expression can be altered by hypoxia, ischaemia or CNS injury [110]. Interestingly, a recent study detected AQP4 expression in the choroid plexus of aged mice, which was undetectable in young mice [148]. They also observed an increase in ventricular size and intraventricular pressure in aged animals when exposed to hypoxia that was less prominent in young animals. This increase in ventricular size was absent in a homogenous AQP4 knockout model. Further, hypoxia induced a cognitive deficit in aged wild-type mice (novel-object recognition test), which was not present in aged AQP4^−/−^ mice. These data implicate AQP4 in hypoxia-induced hydrocephalus and subsequent cognitive decline, likely because of increased CSF secretion. Experiments using AQP knockout mice indicate that osmotically driven water transport following ischaemia or acute water intoxication (leading to cytotoxic oedema) is mediated by AQP4 in the presence of an intact BBB [149]. Following MCAo in mice, AQP4 expression has been shown to be temporarily reduced or lost around 24 h post stroke during the reperfusion phase, and a partial recovery by 72 h post stroke [150]. The authors concluded that the biphasic change in perivascular AQP4 expression might define water influx subsequent to oedema at 24 h, followed by supporting absorption of excess fluid by 72 h. This suggests that perhaps reducing the expression level of perivascular AQP4 is the brain’s own defence mechanism by which it tries to limit water influx via the water channels following ischaemia when BBB integrity is not compromised. In addition, hydrocephalus induced by hypoxia during ischaemic stroke would contribute to an overall elevation in ICP, and if increased CSF secretion does in fact occur in response to hypoxia, then this is an important consideration in current animal models of stroke, particularly MCAo, in which occlusion of the anterior choroidal artery can induce a degree of hypoxia within the choroid plexus. Several attempts have been made to develop pharmacological inhibitors of AQP4 to reduce brain oedema following ischaemia with little success [151]. Recently, Far et al. [152] have shown the effectiveness of two promising AQP4 inhibitors following a high throughput screening of small molecule libraries. AER-270 and AER-271 prevented oedema in a mouse model of water intoxication and in rodent models of MCAo respectively.

## Conclusions

This review summarises our current understanding of CSF dynamics with a focus on effects on ICP during neurological diseases, and highlights some of the discrepancies within the field. The implications of such findings are of high clinical relevance for understanding and treating neurological diseases where brain fluid homeostasis is impaired. Our efforts to elucidate the regulators and mechanisms involved in CSF secretion are still ongoing. Some evidence suggests the involvement of AQP1 at the choroid plexus [83], and more recent discoveries implicate the molecular transfer of water via NKCC1 in CSF secretion [70]; these insights may provide targets for therapeutic control of CSF in conditions of excessive secretion and elevated ICP. Regarding CSF drainage, the conventional view of transport into the superior sagittal sinus by arachnoid projections is challenged with evidence of the involvement of extracranial lymphatics [14–18]. Other hypotheses of CSF drainage have been proposed but are currently criticised within the field. Inhibitors of CSF production, like acetazolamide, are widely used in clinic for lowering ICP, and animal studies have demonstrated their ability to decrease ICP in rats [153, 154]. However, their efficacy in humans is controversial with some reporting attenuated symptoms of elevated ICP in IIH patients prescribed acetazolamide [155], and others reporting that weight loss was more efficacious than acetazolamide [156]. Further, a recent Cochrane review concluded that there is currently insufficient evidence to support or reject the clinical use of acetazolamide in treating symptoms of elevated ICP in IIH [157]. Recent evidence suggests a role for AQPs, TRPV4, and NKCC1 in CSF production. Determining the exact role of these proteins in modulating CSF dynamics and the resulting influence on ICP offers potential to identify therapies that are of translational value. This is extremely important as ICP elevation can lead to secondary neurodegeneration after insult/injury, and in some cases, can even be life threatening.
